# High Contrast PET Imaging of Subcortical and Allocortical Amyloid-β in Early Alzheimer’s Disease Using [^11^C]AZD2184

**DOI:** 10.3233/JAD-231013

**Published:** 2024-04-16

**Authors:** Patrik Mattsson, Zsolt Cselényi, Anton Forsberg Morén, Yvonne Freund-Levi, Lars-Olof Wahlund, Christer Halldin, Lars Farde

**Affiliations:** a Department of Clinical Neuroscience, Centre for Psychiatry Research, Karolinska Institutet and Stockholm Health Care Services, Stockholm, Sweden; b PET Science Centre, Personalized Medicine and Biosamples, R&D, AstraZeneca, Stockholm, Sweden; c Department of Clinical Science and Education, Södersjukhuset, Karolinska Institutet, Stockholm, Sweden; d School of Medicine, Örebro University, Örebro, Sweden; e Department of Geriatrics, Örebro University Hospital, Örebro and Södertälje Hospital, Södertälje, Sweden; f Department of Neurobiology, Care Sciences and Society, Division of Clinical Geriatrics, Center for Alzheimer Research, Karolinska Institutet, Stockholm, Sweden

**Keywords:** Alzheimer’s disease, amygdala, amyloid deposits, entorhinal cortex, hippocampus, positron emission tomography, striatum, thalamus

## Abstract

**Background:**

Deposits of amyloid-β (Aβ) appear early in Alzheimer’s disease (AD).

**Objective:**

The aim of the present study was to compare the presence of cortical and subcortical Aβ in early AD using positron emission tomography (PET).

**Methods:**

Eight cognitively unimpaired (CU) subjects, 8 with mild cognitive impairment (MCI) and 8 with mild AD were examined with PET and [^11^C]AZD2184. A data driven cut-point for Aβ positivity was defined by Gaussian mixture model of isocortex binding potential (*BP*_ND_) values.

**Results:**

Sixteen subjects (3 CU, 5 MCI and 8 AD) were Aβ-positive. *BP*_ND_ was lower in subcortical and allocortical regions compared to isocortex. Fifteen of the 16 Aβ-positive subjects displayed Aβ binding in striatum, 14 in thalamus and 10 in allocortical regions.

**Conclusions:**

Aβ deposits appear to be widespread in early AD. It cannot be excluded that deposits appear simultaneously throughout the whole brain which has implications for improved diagnostics and disease monitoring.

## INTRODUCTION

Brain deposits of amyloid-β (Aβ) appear early in the pathophysiology of Alzheimer’s disease (AD) [1]. On the basis of postmortem studies, the regional appearance of Aβ has been suggested to follow a sequence from cortical to allocortical to subcortical regions [2, 3]. However, postmortem studies typically include brains from patients with advanced AD and the early regional appearance of Aβ requires more detailed understanding.

The development of suitable radioligands for neuroimaging with positron emission tomography (PET) has enabled *in vivo* measurements of Aβ deposits, and several studies have focused on the early appearance of Aβ in cortical regions [4–8]. Recently, Pittsburgh compound B (PIB) and related radioligands have been used to demonstrate Aβ deposition in large subcortical regions such as the striatum [9, 10]. However, detection of Aβ in smaller, low-density regions has been limited by the resolution of the PET systems and the signal-to-noise ratio of the radioligands used.

The Aβ radioligand [^11^C]AZD2184 has been developed to provide higher contrast when compared to other radioligands. [^11^C]AZD2184 has been characterized and evaluated in preclinical studies [11, 12] and in PET studies in control subjects and AD patients [13–16]. In summary, [^11^C]AZD2184 has been shown to share the same binding site to Aβ as the reference radioligand PIB, to have high specific binding and lower degree of non-specific binding compared to that of PIB. These properties enable high contrast Aβ PET imaging with a high-resolution research tomograph (HRRT), which makes [^11^C]AZD2184 suitable for PET measurements in small regions and regions with low levels of Aβ.

In the present PET study we examined cognitively unimpaired (CU) subjects, patients with mild cognitive impairment (MCI), and mild AD with [^11^C]AZD2184 and HRRT. The primary aim was to use high contrast imaging to compare the presence of amyloid deposits in cortical and subcortical regions *in vivo* to confirm and extend the results from previous postmortem studies.

## MATERIALS AND METHODS

### Subjects

The study was approved by the Regional Ethics Committee of Stockholm and the Radiation Safety Committee at the Karolinska University Hospital. It was performed in accordance with the Declaration of Helsinki and the International Conference on Harmonization/Good Clinical Practice guidelines. All subjects gave written informed consent before participation in the study. The study was conducted at the PET center, Karolinska University Hospital, Solna, during 2009–2015.

In total, 24 subjects were included. Eight CU subjects, 8 MCI, and 8 mild AD patients were recruited from the memory clinics of Karolinska University Hospital, Jakobsberg Hospital and Dalen Hospital in the Stockholm region. CU subjects were recruited from a cohort of control subjects that were originally identified among volunteers from a senior citizen’s association, spouses of memory clinic patients, and subjects with subjective memory complaints. The CU subjects had no cognitive deficits according to cognitive assessment performed at the memory clinic of Karolinska University Hospital before inclusion in the present study. MCI and AD patients had been diagnosed at the memory clinics according to DSM-IV criteria [17]. MCI patients with amnestic or non-amnestic presentation were eligible for inclusion. Genotyping for *APOE* was performed in 11 subjects prior to inclusion in the present study. None of the patients had been exposed to anti-Aβ monoclonal antibodies.

Global cognitive function was assessed with Mini-Mental State Examination (MMSE) [18] and clinical severity was assessed with Global Deterioration Scale (GDS) [19] when the subjects were included in the present study (assessments performed by PM). Mild AD was defined as MMSE ≥20. GDS has seven stages, where stages 1–3 are predementia stages and stages 4–7 are dementia stages. Saliva samples were collected using Oragene·DNA OG-500 (DNA Genotek Inc., Canada, www.dnagenotek.com) from 13 subjects who had not previously been tested for *APOE*. The saliva samples were analyzed for *APOE* genotype at the Karolinska University Laboratory.

### MRI

T1-weighted MR images were acquired with 1.5 T MRI systems at Karolinska University Hospital and used for coregistration with PET, as well as for delineation of regions of interest (ROIs).

### PET experimental procedure

An individual plaster helmet was made for each subject prior to PET and used during the PET measurement to minimize head movements [20]. Radiosynthesis of [^11^C]AZD2184 was performed according to a procedure described previously [12]. The mean radioactivity injected i.v. and the mean molar activity was 426 (365–508) MBq and 270 (102–590) GBq/*μ*mol, respectively, for the controls. The corresponding values for the MCI patients were 383 (239–518) MBq and 171 (80–363) GBq/*μ*mol, and for the AD patients 474 (381–518) MBq and 266 (53–556) GBq/*μ*mol.

Brain radioactivity was measured for 63 min. PET data acquisition was performed with a high-resolution research tomograph (HRRT, CTI/Siemens, Knoxville, TN, USA). Data were acquired continuously in list mode and reconstructed into 33 time frames according to the sequence: 9×10 s, 2×15 s, 3×20 s, 4×30 s, 4×60 s, 4×180 s, and 7×360 s. The in-plane resolution of the HRRT system at the PET center, Karolinska University Hospital, Solna, is 1.5 mm full-width at half-maximum (FWHM) in the center of the field of view (FOV) and 2.4 mm at 10 cm off-center directions [21].

### PET image analysis

PET image analysis was performed using an in-house processing pipeline (Solena) running in MATLAB (R2014b) and FreeSurfer (v. 5.0.0, http://surfer.nmr.mgh.harvard.edu/). The software used SPM5 for image conversion and spatial processing steps. ROIs were defined for frontal cortex, occipital cortex, parietal cortex, temporal cortex, anterior cingulate cortex, posterior cingulate cortex, insula, parahippocampal cortex, sensorimotor cortex, striatum, thalamus, amygdala, entorhinal cortex, hippocampus, and white matter using FreeSurfer (version 5.0.0). The choice of ROIs was based on the suggested sequence of regional appearance of Aβ from cortical to subcortical regions [3]. White matter was included as a ROI since Aβ has been observed in subcortical white matter in addition to deposition in grey matter [2]. The cerebellar cortex was used as reference region for free and non-specifically bound [^11^C]AZD2184 [15]. The cerebellar cortex was trimmed, using only voxels above the lowest plane of the pons, behind and at/below the posterior tip of the fourth ventricle.

### Quantification

Quantification was performed using wavelet aided parametric imaging (WAPI), which employs the multi-linear variant of the reference region-based Logan graphical analysis to estimate the binding potential (*BP*_ND_) for each voxel. The background and procedure for the wavelet-based analysis has previously been described in detail [22]. ROIs were applied to the individual parametric images and regional *BP*_ND_ was calculated as the average voxel value inside each ROI.

### Isocortex ROI definition

Volume-weighted averages of frontal cortex, occipital cortex, parietal cortex, temporal cortex, anterior cingulate cortex, posterior cingulate cortex, sensorimotor cortex, insula and parahippocampal cortex *BP*_ND_ were calculated for each participant. The resulting isocortex *BP*_ND_ values were used for Aβ threshold definition. Isocortex *BP*_ND_ were also used for comparison with *BP*_ND_ in the allocortical and subcortical regions (entorhinal cortex, hippocampus, amygdala, striatum and thalamus). The choice of isocortex (rather than the individual cortical regions) for comparison was based on the suggested sequence of regional appearance of Aβ [3].

### Aβ threshold definition

A Gaussian mixture model (GMM) with 2 components was used as an approach to fit the isocortex *BP*_ND_ values to a low Aβ and a high Aβ distribution. The results of the GMM assign each subject a posterior probability of belonging to a distribution. A cut-off between the low Aβ and the high Aβ distributions was derived from the intersection of the density curves of the two distributions. The low Aβ group was considered Aβ-negative and the high Aβ group was considered Aβ-positive.

### Descriptive analysis

In the Aβ-positive subjects, the appearance of subcortical Aβ was evaluated according to a procedure described in the postmortem literature [3]. This approach relies on the assumption that regions displaying Aβ in a majority of cases are regions with early-appearing Aβ, whereas regions displaying Aβ in fewer cases are regions with late-appearing Aβ. Specifically, the number of cases displaying Aβ was noted for each region. The regions were sorted in descending order from higher frequency to lower frequency Aβ regions. Then, for each case, the presence of Aβ in a region that displayed Aβ in more cases (higher frequency Aβ region) was compared with the presence of Aβ in a region that displayed Aβ in fewer cases (lower frequency Aβ region). A hierarchical sequence of Aβ appearance was assumed if cases with Aβ in a lower frequency Aβ region consistently displayed Aβ in a higher frequency Aβ region. The assumption of a hierarchical sequence was not supported if a case with Aβ in a lower frequency Aβ region did not display Aβ in a higher frequency Aβ region.

### Statistical analysis

All statistical analyses were performed with the software environment R, version 4.2.1 (R Core Team (2021). URL https://www.R-project.org/). T-test and analysis of variance (ANOVA) were used to compare means, and Fisher’s exact test was used to compare categorical observations. Pearson’s *r* was calculated for correlations between subcortical and isocortex *BP*_ND_. Linear regression was used to estimate slopes and intercepts for the regression lines (subcortical *BP*_ND_ regressed on isocortex *BP*_ND_). Specifically, regression slopes were compared in an approach to evaluate differences in rate of Aβ deposition, and intercepts were compared in an approach to evaluate whether regional Aβ deposition occurs before or after Aβ deposition in isocortex.

## RESULTS

### Demographical and clinical characteristics of subjects

Twenty-four subjects (8 CU subjects, 8 MCI patients, 8 AD patients) participated according to the protocol. Demographics and clinical characteristics are presented in Table 1. No distinction was made between amnestic and non-amnestic MCI. There were no statistically significant group differences in mean age (*F*(2,21) = 0.12, *p* = 0.88), sex (Fisher’s exact test, *p* = 0.89) or *APOE4* carriership (Fisher’s exact test, *p* = 0.06). Mean MMSE differed significantly between groups (*F*(2,21) = 4.55, *p* = 0.02), and *post hoc* analysis showed a significant difference between CU subjects and AD patients, with an adjusted *p* = 0.02 (Tukey multiple comparisons of means).

**Table 1:** Demographic data and Mini-Mental State Examination (MMSE)

	CU (*n* = 8)	MCI (*n* = 8)	AD (*n* = 8)	Total (*n* = 24)
Age, mean (range)	73.2 (60–84)	71.9 (64–81)	73.5 (63–83)	72.9 (60–84)
Sex, female/male	4/4	4/4	5/3	13/11
*APOE4*, carrier/noncarrier	2/6	4/4	7/1	13/11
MMSE, mean (range)	29.0 (28–30)	27.6 (24–30)	25.4 (20–30)	27.3 (20–30)

Clinical severity, assessed with GDS, ranged from 1 (no cognitive decline) to 4 (mild dementia) in the total sample, with a mode of 1 in CU subjects, 3 in MCI patients and 4 in AD patients. There was, however, some overlap in clinical severity between groups, with GDS 2 (subjective complaints/very mild cognitive decline) in 1 CU subject and 2 MCI patients, and GDS 3 (mild cognitive decline) in 1 AD patient.

### Time-activity curves

**Fig. 1:**
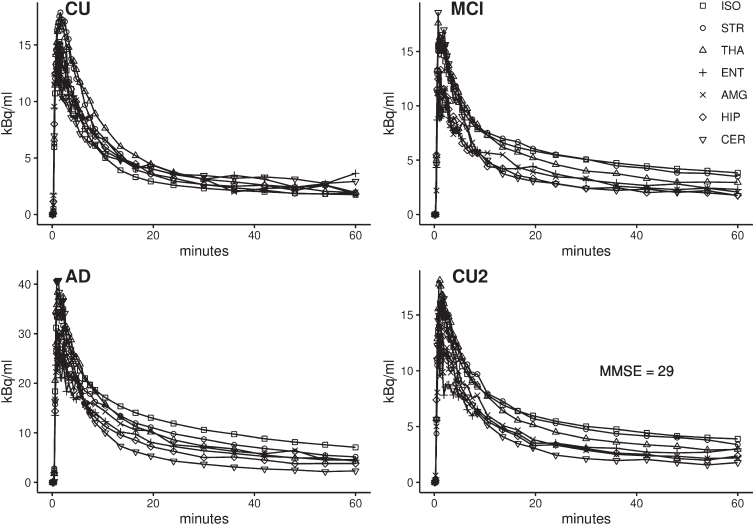
Time activity curves (TAC) of [^11^C]AZD2184 in a cognitively unimpaired subject (CU), a mild cognitive impairment (MCI) patient, an Alzheimer’s disease (AD) patient and a CU subject with higher radioactivity concentration in all target regions compared to the reference region (CU2). ISO, isocortex; STR, striatum; THA, thalamus; ENT, entorhinal cortex; AMG, amygdala; HIP, hippocampus; CER, cerebellum.

Representative time-activity curves for a CU subject, an MCI patient and an AD patient are presented in Fig. 1. Following i.v. injection of [^11^C]AZD2184, there was a rapid increase in regional brain radioactivity followed by a rapid early decline in target regions (isocortex, striatum, thalamus, entorhinal cortex, amygdala, hippocampus) as well as in the reference region (cerebellar cortex). In addition, one CU subject had higher radioactivity concentration in all target regions compared to the reference region, resembling the time-activity curve patterns of the MCI and AD patients (Fig. 1).

### Regional Aβ binding in the total sample

Regional *BP*_ND_ values were derived from wavelet aided parametric images. Mean *BP*_ND_ was highest in isocortex, intermediate in striatum and thalamus, and low in entorhinal cortex, amygdala, and hippocampus (Table 2). In the total sample some individual *BP*_ND_ values were negative, i.e., radioactivity in the cerebellum was slightly higher than in the ROI.

**Table 2:** Regional *BP*_ND_ values for [^11^C]AZD2184 binding in the total sample and Aβ-positive subsample

ROI	Total sample (*n* = 24)		Aβ-positive (*n* = 16)	
	mean	(range)	Mean	(range)
Isocortex	0.50	(–0.16–1.41)	0.75	(0.25–1.41)
Striatum	0.35	(–0.17–1.02)	0.54	(0.11–1.02)
Thalamus	0.31	(–0.08–0.82)	0.41	(0.04–0.82)
Entorhinal cortex	0.08	(–0.29–0.37)	0.17	(0.00–0.37)
Amygdala	0.04	(–0.46–0.45)	0.14	(–0.17–0.45)
Hippocampus	–0.02	(–0.44–0.20)	0.05	(–0.24–0.20)
White matter	0.21	(–0.07–0.48)	0.24	(–0.04–0.48)

### Aβ threshold identification

**Fig. 2:**
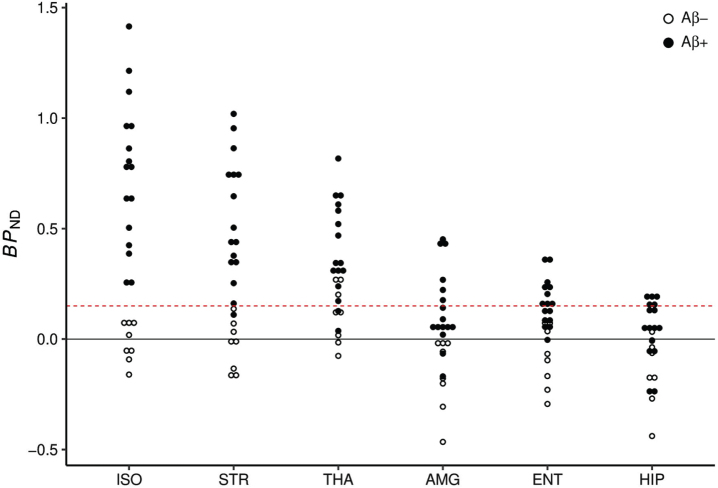
Individual [^11^C]AZD2184 *BP*_ND_ values for Aβ-negative (Aβ–) and Aβ-positive (Aβ+) subjects. The dashed line indicates threshold for Aβ positivity (0.15). ISO, isocortex; STR, striatum; THA, thalamus; ENT, entorhinal cortex; AMG, amygdala; HIP, hippocampus.

AZD2184 binding in isocortex was used as a global cortical measure of Aβ deposition to identify a cut-off between Aβ-negative and Aβ-positive subjects. Fitting a bimodal distribution to the isocortex *BP*_ND_ values by GMM assigned 8 subjects (5 CU, 3 MCI) to the Aβ-negative group and 16 subjects (3 CU, 5 MCI, 8 AD) to the Aβ-positive group, and the thereby identified global isocortex *BP*_ND_ cut-off was 0.15 (Fig. 2). Notably, all Aβ-negative subjects had isocortex *BP*_ND_≤0.08, and all Aβ-positive subjects had isocortex *BP*_ND_≥0.25. Mean isocortex *BP*_ND_ for Aβ-negative subjects was close to 0 (–0.01, range –0.16 –0.08) and similar to mean grey matter binding in young and elderly CU subjects in previous studies with [^11^C]AZD2184 [13, 16].

### Regional Aβ binding in Aβ-positive subjects

Regional mean *BP*_ND_ values for Aβ-positive subjects are presented in Table 2. Mean *BP*_ND_ was highest in isocortex, intermediate in striatum (72% of isocortex) and thalamus (55% of isocortex), and low in entorhinal cortex (23% of isocortex), amygdala (19% of isocortex), and hippocampus (7% of isocortex). There was, however, a large individual variation in *BP*_ND_ across regions. Similar *BP*_ND_ were, e.g., observed in isocortex and striatum, in isocortex and thalamus, and in isocortex, striatum and thalamus in some subjects. In entorhinal cortex, amygdala, and hippocampus, individual *BP*_ND_ values were generally below 50% of isocortex *BP*_ND_. However, in some subjects, *BP*_ND_ in entorhinal cortex, amygdala, and hippocampus ranged between 52–59% of isocortex *BP*_ND_.

Fifteen of the Aβ-positive subjects had [^11^C]AZD2184 binding above the threshold in striatum, 14 in thalamus, 9 in entorhinal cortex, 6 in amygdala, and 5 in hippocampus (Fig. 2). A majority of Aβ-positive subjects thus displayed [^11^C]AZD2184 binding above threshold in striatum, thalamus, and entorhinal cortex.

A hierarchical sequence of apparently earlier Aβ appearance in isocortex (Aβ-positive in all cases by definition) could be described for the pairwise comparisons between isocortex and all other regions, however the differences in the numbers of observations were small, especially for striatum and thalamus.

Pairwise comparison of striatum and thalamus further showed that, although there were more cases with Aβ in striatum than cases with Aβ in thalamus, there was no consistent hierarchical sequence in Aβ appearance between the two regions (1 case displayed Aβ in thalamus but not in striatum). Aβ could thus be displayed in striatum or thalamus in all 16 Aβ-positive subjects.

In addition, although there were more cases with Aβ in entorhinal cortex than in hippocampus, no consistent hierarchy in Aβ appearance could be discerned between these two regions either (1 case displayed Aβ in hippocampus but not in entorhinal cortex). Aβ could thus be displayed in allocortical regions (entorhinal cortex or hippocampus) in 10 of the Aβ-positive subjects.

Mean white matter [^11^C]AZD2184 binding was slightly higher in Aβ-positive subjects compared to the total sample (Table 2), and 60% higher (mean *BP*_ND_ = 0.24) than in Aβ-negative subjects (mean *BP*_ND_ = 0.15). The difference between Aβ-positive and Aβ-negative subjects was, however, statistically non-significant (*p* = 0.21).

Another observation was that, in contrast to the separation of [^11^C]AZD2184 binding in isocortex into an Aβ-negative and an Aβ-positive group, no clear separation between the Aβ-negative and Aβ-positive groups could be observed in the subcortical regions (Fig. 2). Instead, [^11^C]AZD2184 binding in the Aβ-negative and Aβ-positive groups overlapped in subcortical regions, primarily because of *BP*_ND_ values below the threshold in some Aβ-positive subjects. In addition, *BP*_ND_ values above the threshold were observed in thalamus and in white matter in some of the Aβ-negative subjects.

### Correlations between regional and isocortex [^11^C]AZD2184 binding

**Fig. 3:**
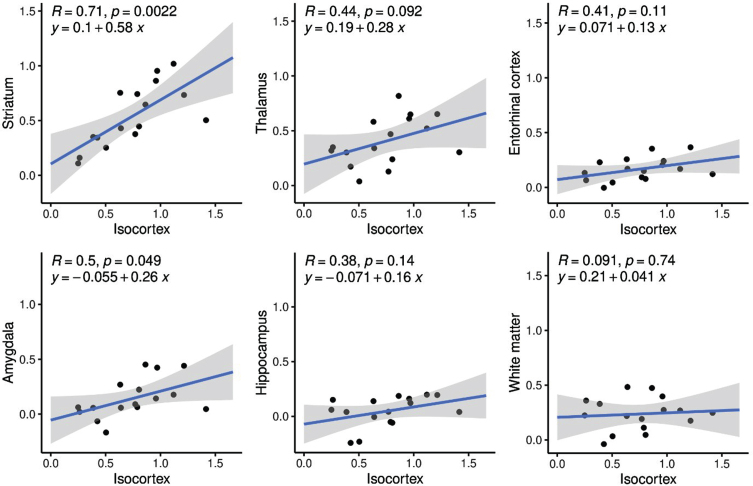
Correlations between [^11^C]AZD2184 *BP*_ND_ in isocortex and subcortical/allocortical regions.

In Aβ-positive subjects, correlations between subcortical and isocortex *BP*_ND_ were positive and statistically significant for the correlations between isocortex and striatum, and isocortex and amygdala, and a trend-level significant correlation was obtained between isocortex and thalamus (Fig. 3). The correlation between isocortex and striatum remained statistically significant after Bonferroni correction for multiple comparisons (i.e., 6 comparisons). The slopes of the regression lines were numerically smaller than 1 for all comparisons and statistically significant for striatum, suggesting slower rate of Aβ accumulation in striatum compared to isocortex (Fig. 3). Confidence intervals for the intercepts overlapped 0 for all comparisons, suggesting no difference in Aβ appearance between regions (Fig. 3).

## DISCUSSION

### General remarks

The aim of this PET study was to compare the presence of Aβ in cortical and subcortical regions using a PET system with the highest resolution available and an Aβ radioligand with the best signal-to-background ratio to our knowledge. Hence, we were able to study regional distribution of Aβ load across patients with varying disease states. Sixteen out of 24 elderly subjects included in the analysis were Aβ-positive, as defined by cortical deposits, and ranged in clinical severity from no cognitive decline to mild dementia. Importantly, Aβ was detected in subcortical and allocortical regions in a majority of Aβ-positive subjects; however, there was some variation in the regional appearance.

### Aβ in striatum

A main finding was that Aβ binding could be detected in striatum in all but one Aβ-positive subject. Severe involvement of Aβ pathology in striatum has previously been observed in AD patients in postmortem studies, as well as the presence of smaller amounts of Aβ in some non-demented subjects [2, 23]. The appearance of Aβ in striatum in full dementia spectrum (CU, MCI, AD) samples has recently been studied with PIB and florbetapir [9, 10]. Even if these studies included larger samples than the present study, the proportions of CU subjects and early AD (MCI+mild AD) patients are comparable. Our results show higher proportions of subjects with Aβ in striatum when compared with total samples, diagnostic groups (CU, MCI, and AD) and, importantly, Aβ-positive subjects in the mentioned studies with PIB and florbetapir.

### Aβ in thalamus

Thalamus is a major subcortical region, consisting of 23 subnuclei and having functionally important connections with regions that are affected in early AD [24, 25]. Postmortem examination of severe AD cases has shown amyloid deposits in almost all thalamic nuclei, whereas control cases had no or only small amounts of amyloid deposits [26]. A slight increase in PIB uptake was observed in mild-to-moderate AD compared to controls in an initial PET study [27]. Little attention has, however, been given to the appearance of Aβ in thalamus in early AD.

Aβ was detected in thalamus in 14 out of the 16 Aβ-positive subjects in the present study, including 3 CU subjects. In addition, Aβ above the threshold was detected in thalamus in 3 Aβ-negative CU subjects. This finding differs from a recent PET study with florbetapir, where none of the CU participants displayed Aβ in thalamus [10]. Frequencies of MCI and AD patients with Aβ in thalamus were not reported in the florbetapir study, precluding further comparison with the present study.

### Aβ in amygdala

Amygdala has, like thalamus, attracted relatively little attention in early AD. Single Aβ deposits begin to appear in amygdala after the appearance of Aβ in isocortex and allocortex, and before the appearance of Aβ in thalamus and striatum according to postmortem findings [3]. Little is, however, known about the appearance of Aβ in amygdala *in vivo*. We were able to detect Aβ in 6 subjects (equal to one fourth of all subjects) in the present study. This is higher than the “involvement” of amygdala in 16% of all subjects in a PET study with florbetaben that included comparable proportions of CU, MCI, and AD in the analysis [5].

### Aβ in allocortical regions

Deposition of Aβ was detected in entorhinal cortex in 9 subjects and in hippocampus in 5 subjects in the present study. According to postmortem studies, small amounts of Aβ appear in allocortex after the appearance of Aβ in isocortical regions, and before Aβ deposits begin to appear in striatum and thalamus [2, 3]. This sequence of Aβ appearance has thus far not been replicated *in vivo*. The reason for this discrepancy is probably due to the lower sensitivity of PET compared to histological methods, which allow for detection of single Aβ deposits. Although the apparent sequence of Aβ deposition did not place allocortex directly after isocortex in the present study, we were able to detect Aβ in allocortex in a majority of the Aβ-positive subjects.

### Aβ in entorhinal cortex

Aβ deposits begin to appear in entorhinal cortex while amounts of Aβ are still low in isocortex and hippocampus is still devoid of Aβ [2, 28, 29]. We were able to detect Aβ in entorhinal cortex in a majority of Aβ-positive subjects (and in more than one third of all subjects) in the present study. This can be compared to the “involvement” of entorhinal cortex in 18% of all subjects in the PET study with florbetaben [5].

### Aβ in hippocampus

Hippocampus has, for a long time, attracted particular attention because it has a central role in episodic memory. It is affected early by Aβ deposits according to postmortem studies, but only after Aβ appears in isocortex and in the entorhinal region [2, 28, 29]. Hippocampus remains mildly involved with relatively few Aβ deposits while deposits continue to increase in isocortex [2, 30]. Despite this, we were able to detect Aβ in 5 subjects in the present study, translating into 21% of the total sample which is a considerably higher proportion of subjects with Aβ than the 1.5% of all subjects with “involved” hippocampus in the florbetaben study [5].

### Radioligand binding in white matter

Mean [^11^C]AZD2184 binding in total white matter was 60% higher than that in the Aβ-negative group; however, the difference was not statistically significant. The binding of Aβ radioligands to white matter has been discussed over the years and is generally considered to mainly reflect non-specific binding [31, 32]. However, Aβ deposits have been observed in postmortem white matter [2], and higher white matter binding has been found in Aβ-positive subjects than in Aβ-negative subjects in other PET studies using PIB and florbetapir [33, 34]. Moreover, soluble Aβ was reported to be frequent in subcortical white matter and not related to the level of Aβ deposits in cortex [35], suggesting that Aβ deposition in white matter and cortex might not be correlated. Although statistically non-significant, it cannot be excluded that the higher white matter [^11^C]AZD2184 binding in Aβ-positive subjects in the present study partly reflects specific binding.

### Correlations and regression analysis

[^11^C]AZD2184 binding in striatum was significantly correlated to the binding in isocortex. The correlations between isocortex *BP*_ND_ and the other regions were weaker and did not reach statistical significance in our small sample. The slopes of the regression lines were below 1, which is in line with a previous study [36]. This can be interpreted as slower rate of Aβ deposition in striatum, thalamus, amygdala, entorhinal cortex, and hippocampus than in isocortex. Intercepts did not differ significantly from zero, suggesting the possibility that subcortical and allocortical Aβ appear in parallel with isocortical Aβ.

### Summary

A clear pattern of [^11^C]AZD2184 binding in subcortical and allocortical regions compared to isocortex could not be observed in our sample. Whereas the timing of Aβ deposition cannot truly be answered in a cross-sectional study, it cannot be excluded that Aβ appears globally in early AD. The apparent regional differences may represent more complicated processes involving different threshold/plateau levels and different rates of Aβ accumulation/clearance, and not propagation from cortical to subcortical regions. Furthermore, choosing isocortex to define Aβ positivity inevitably biases towards the assumption of isocortex being earlier than other regions. However, worth noting is that we observed [^11^C]AZD2184 binding above threshold in thalamus in some subjects that were Aβ-negative in isocortex. Interestingly, a recent study in CU and MCI has suggested the possibility of Aβ accumulation starting either in cortical regions or in subcortical regions [37], thus further challenging the established view of the early appearance of Aβ.

### Limitations

One obvious limitation of the present study is the small sample size. The results need to be replicated in longitudinal studies of larger samples with high contrast PET, before firmer conclusions can be drawn about the regional appearance and rate of Aβ deposition.

The use of clinical diagnostic criteria (without biomarker evidence of AD) is another limitation of the present study. However, all AD patients and a major proportion of the MCI patients were Aβ-positive. Since our main aim was to compare the presence of amyloid deposits in cortical and subcortical regions in a combined group of CU, MCI and AD, we do not think that overlap between diagnostic groups has affected the results.

The lower non-specific binding of [^11^C]AZD2184, compared to that of PIB, resulting in a several-fold higher signal-to-background ratio, enabled us to detect Aβ in low-density regions such as entorhinal cortex and hippocampus in several subjects. However, the sensitivity of [^11^C]AZD2184 may still not be sufficiently high for detection in patients with very low levels of Aβ in allocortical regions. This interpretation is supported by a large postmortem study of regional plaque counts, showing low number of plaques in allocortical regions compared to association cortices throughout the suggested phases of Aβ deposition [30].

The relatively low resolution of PET can further lead to partial volume effects (PVEs), especially in small regions, resulting in lower signal due to “spill out” of radioactivity to adjacent white matter and cerebrospinal fluid spaces. The high-resolution PET system used in the present study, however, increases the recovery of signal in the ROI and thus reduces PVE. In addition, low Aβ density regions are sensitive to noise, which is illustrated by negative binding potential values in some small regions for some individuals. In summary, we cannot exclude the possibility that the number of subjects positive for Aβ in allocortical regions was underestimated due to limits of detection of very low densities of Aβ.

### Conclusions

We conclude that Aβ deposits appear to be widespread in cortical and subcortical regions in early AD. We suggest that high contrast PET can be used for detailed study of the early appearance of Aβ, and that it has potential for improved diagnostics and monitoring of disease-modifying treatments.

## AUTHOR CONTRIBUTIONS

Patrik Mattsson (Conceptualization; Formal analysis; Investigation; Project administration; Visualization; Writing – original draft); Zsolt Cselényi (Conceptualization; Software; Writing – review & editing); Anton Forsberg Morén (Conceptualization; Project administration; Writing – review & editing); Yvonne Freund-Levi (Resources; Writing – review & editing); Lars-Olof Wahlund (Conceptualization; Resources; Supervision; Writing – review & editing); Christer Halldin (Conceptualization; Resources; Supervision; Writing – review & editing); Lars Farde (Conceptualization; Funding acquisition; Resources; Supervision; Writing – review & editing).

## Data Availability

The data supporting the findings of this study are available on reasonable request from the corresponding author.
